# Supplementary material for: Investigating the Impact of Dietary Fibers on Mycotoxin Bioaccessibility during In Vitro Biscuit Digestion and Metabolites Identification
Source: Foods. 2023 Aug 23;12(17):3175. doi: 10.3390/foods12173175 (PMC10486935; doi:10.3390/foods12173175)
Supplement: Supplementary file 1 [file foods-12-03175-s001.zip › foods-2566346-supplementary.pdf]

Investigating the Impact of Dietary Fibers on Mycotoxin Bioaccessibility during In Vitro Biscuit Digestion and Metabolites Identification

Rosalía López-Ruiz [1,2,*], Jesús Marin-Saez [1,2], Sara C. Cunha [1], Ana Fernandes [3], Victor de Freitas [3], Olga Viegas [1,4] and Isabel M. P. L. V. O. Ferreira [1]

[1] LAQV/REQUIMTE, Laboratory of Bromatology and Hydrology, Department of Chemical Sciences, Porto University, 4050-313 Porto, Portugal; jms485@ual.es (J.M.-S.); sara.cunha@ff.up.pt (S.C.C.); olgaviegas@fcna.up.pt (O.V.); isabel.ferreira@ff.up.pt (I.M.P.L.V.O.F.)
[2] Research Group "Analytical Chemistry of Contaminants", Department of Chemistry and Physics, Research Centre for Mediterranean Intensive Agrosystems and Agri-Food Biotechnology (CIAIMBITAL), University of Almeria, Agrifood Campus of International Excellence, ceiA3, E-04120 Almeria, Spain
[3] LAQV/REQUIMTE, Department of Chemistry and Biochemistry, Science Faculty, Porto University, 4169-007 Porto, Portugal; ana.fernandes@fc.up.pt (A.F.); vfreitas@fc.up.pt (V.d.F.)
[4] Faculty of Nutrition and Food Sciences, University of Porto, 4150-180 Porto, Portugal
* Correspondence: rosalialr@ual.es

Table of contents

Table S1. Moisture, fibre and carbohydrates of apple flours (n=2).

Table S2. L*a*b* color space results for biscuits containing dietary fibre and ΔE (n=4).

Table S1. Moisture, fibre and carbohydrates of apple flours (n=2).

Sample	Moisture (%)	Insoluble fibre (%)	Soluble fibre (%)	Total dietary fibre content (%)	Carbohydrates (%)
**FS**	2.7	21.5	7.2	28.7	64.9 (55% fructose; 24% glucose; 21% sucrose)
**FP**	7.2	53.2	15.9	69.1	19.6 (70% fructose; 9% glucose; 21% sucrose)

Abbreviations: FS: apple flour with sugars; FP: apple flour without sugar powered

Table S2. L*a*b* color space results for biscuits containing dietary fibre and ΔE (n=4).

**Sample**	**L***	**a***	**b***	**ΔE# sample vs control**
**Control**	55.11	4.00	23.95	
**A**	52.54	5.03	23.23	2.86
**K**	52.01	5.42	24.00	3.41
**PC**	51.11	5.45	23.24	4.30
**FS**	49.84	8.48	32.93	5.25
**FP**	58.28	6.12	35.21	11.93

Abbreviations: A: arabinogalactan; K: k-carrageenan; PC: commercial pectin; FS: apple pomace flour with sugars; FP: powered apple pomace flour without sugar.
